# Automated analysis of computerized morphological features of cell clusters associated with malignancy on bile duct brushing whole slide images

**DOI:** 10.1002/cam4.5365

**Published:** 2022-10-24

**Authors:** Shayan Monabbati, Patrick Leo, Kaustav Bera, Claire W. Michael, Behtash G. Nezami, Aparna Harbhajanka, Anant Madabhushi

**Affiliations:** ^1^ Department of Biomedical Engineering Case Western Reserve University Cleveland Ohio USA; ^2^ Department of Pathology Case Western Reserve University School of Medicine, University Hospitals Cleveland Medical Center Cleveland Ohio USA; ^3^ Louis Stokes Cleveland Veterans Administration Medical Center Cleveland Ohio USA

**Keywords:** bile duct brushings, biliary tract adenocarcinoma, computer‐aided diagnosis, digital pathology, machine learning

## Abstract

**Background:**

Bile duct brush specimens are difficult to interpret as they often present inflammatory and reactive backgrounds due to the local effects of stricture, atypical reactive changes, or previously installed stents, and often have low to intermediate cellularity. As a result, diagnosis of biliary adenocarcinomas is challenging and often results in large interobserver variability and low sensitivity

**Objective:**

In this work, we used computational image analysis to evaluate the role of nuclear morphological and texture features of epithelial cell clusters to predict the presence of pancreatic and biliary tract adenocarcinoma on digitized brush cytology specimens.

**Methods:**

Whole slide images from 124 patients, either diagnosed as benign or malignant based on clinicopathological correlation, were collected and randomly split into training (*S*_T_, *N* = 58) and testing (*S*_*v*_, *N* = 66) sets, with the exception of cases diagnosed as atypical on cytology were included in *S*_*v*_. Nuclear boundaries on cell clusters extracted from each image were segmented via a watershed algorithm. A total of 536 quantitative morphometric features pertaining to nuclear shape, size, and aggregate cluster texture were extracted from within the cell clusters. The most predictive features from patients in *S*_T_ were selected via rank‐sum, *t*‐test, and minimum redundancy maximum relevance (mRMR) schemes. The selected features were then used to train three machine‐learning classifiers.

**Results:**

Malignant clusters tended to exhibit lower textural homogeneity within the nucleus, greater textural entropy around the nuclear membrane, and longer minor axis lengths. The sensitivity of cytology alone was 74% (without atypicals) and 46% (with atypicals). With machine diagnosis, the sensitivity improved to 68% from 46% when atypicals were included and treated as nonmalignant false negatives. The specificity of our model was 100% within the atypical category.

**Conclusion:**

We achieved an area under the receiver operating characteristic curve (AUC) of 0.79 on *S*_*v*_, which included atypical cytological diagnosis.

## Graphical abstract

Pancreatobiliary adenocarcinomas can be diagnosed with a computerized approach with sensitivity of 63%. Atypical cases can be diagnosed with 100% specificity.

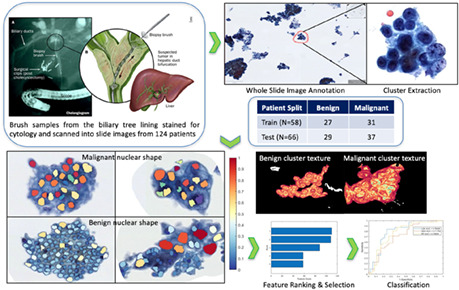

## 1 INTRODUCTION

Bile duct brushings (BDBs) are the preferred method of screening for pancreatic adenocarcinoma and cholangiocarcinoma due to their relative ease of accessibility of bile duct lesions and low complication rate compared with biopsies.^1^ A BDB procedure involves inserting a brush attached to a catheter, which samples the epithelium lining in the bile duct stricture, which is a narrowing in the lumen of the bile duct potentially caused by either primary malignancy or invasion occurring in the pancreas or elsewhere in the biliary tree.^1^ The common etiologies of strictures include infectious processes, intrabiliary lithiasis, adenocarcinoma, or previously installed stents.^2^ As a result, the classification of cell clusters on BDBs is extremely challenging. Cell clusters that present as such are sometimes classified as atypical.^2^

The diagnosis of atypical cell clusters often results in large interobserver variability and low sensitivity (6–64%, mean 42%).^3^ It is important to diagnose these cases accurately for appropriate management including chemotherapy and/or surgery (i.e. Whipple procedures).^4^ The sensitivity of cytology can be improved with the use of adjunctive tests such as digital image analysis, assessment of KRAS mutation status, and fluorescent in situ hybridization (FISH) for polysomy.^5^, ^6^ In particular, the sensitivity of digital image analysis varies between 14% and 48% and is only slightly increased when combined with FISH.^5^, ^6^ A study investigating the quantitative PCR for KRAS mutation showed an improvement of sensitivity of 47%, however, its analysis was restricted to a single gene, potentially limiting the sensitivity of the test.^4^ Multicolor FISH tests performed using the UroVysion probe set (Abbott Molecular Inc.) have achieved sensitivity ranging from 35% to 60%.^7^ These tests are labor intensive, costly, and difficult to interpret due to overlapping nuclei, which further provides clinical reasons to develop new modalities to improve diagnostic sensitivity on cytology of biliary tract adenocarcinomas.

Multiple recuts can be obtained from paraffin‐embedded tissue blocks from the same patient in case slides get damaged. This naturally allows for a larger quantity of morphological data to be obtained from each patient. However, in cytology, every slide is irreplaceable, which means that there exists a unique set of morphological features for any given slide. This limitation, on top of the cost of modern imaging modalities, are a few reasons why there is a lack of digital cytopathological data available worldwide.^8^ Particularly given the limited cases of biliary adenocarcinoma, deep‐learning (DL) model would likely not have been suitable for such a study due to the limited training data available for developing an accurate DL model for this problem. DL networks in this field have attempted to segment nuclei and cytoplasm from cervical cells to learn the morphological differences between benign and malignant samples.^9^, ^10^ Zhang *et al*.^11^ were able to classify benign and malignant cervical cell samples with an accuracy of 98%, however, current models avoid the handcrafted‐feature approach potentially due to the lack of biological explainability of stable diagnostic signals. These DL models require a large amount of training data to make reliably accurate predictions and lack the clinical interpretability of the driving signals of successful diagnosis and hence why they lack application in practice. Moreover, algorithms that are able to refer the cytopathologist to a digital collection of relevant cell clusters visible in large magnification on a computer are more timely than having to review individual slide images under a microscope.^12^ Review articles and studies, such as Boroujeni et al,^13^ have discussed different imaging modalities used to analyze morphological patterns for pancreatic and pancreatobiliary adenocarcinomas,^9^, ^10^ however, no study has attempted to characterize morphological imaging markers on cytology specimens using a clinically interpretable and automated method.

Several studies have attempted to delineate definite, handcrafted cytological criteria aimed to predict malignancy in BDBs. In 1995, Cohen *et al*. were the first to use cytological criteria to improve diagnostic sensitivity.^14^ They found that nuclear molding, chromatin clumping, and increased nuclear‐cytoplasmic (N:C) ratio were frequently associated with malignancy (deemed ‘primary Iowa criteria’). These perinuclear features have been shown to differentiate lung cancer subtypes through the use of image texture.^15^ Although textural analysis has not yet been performed on cytopathology in the context of biliary adenocarcinoma, these perinuclear features could potentially be captured by quantifying the texture around nuclear boundaries and within the cytoplasm. If these “primary criteria” were not present, a set of secondary criteria, which included anisonucleosis, nuclear irregularity/grooves, and nuclear enlargement were used. The presence of 2/3 primary criteria yielded 83% sensitivity and 98% specificity for the detection of adenocarcinoma. In 1998, Renshaw *et al*. similarly found that nuclear molding, chromatin clumping, and loss of cellular polarity were associated with malignancy (deemed the “Boston criteria”).^16^ In particular reference to whole slide image (WSI) analysis, Collins *et al*. evaluated a number of computational features on WSIs such as individual nuclear area, the number of nuclei per group, N:C ratio, and nuclear size differential.^17^

**FIGURE 1:**
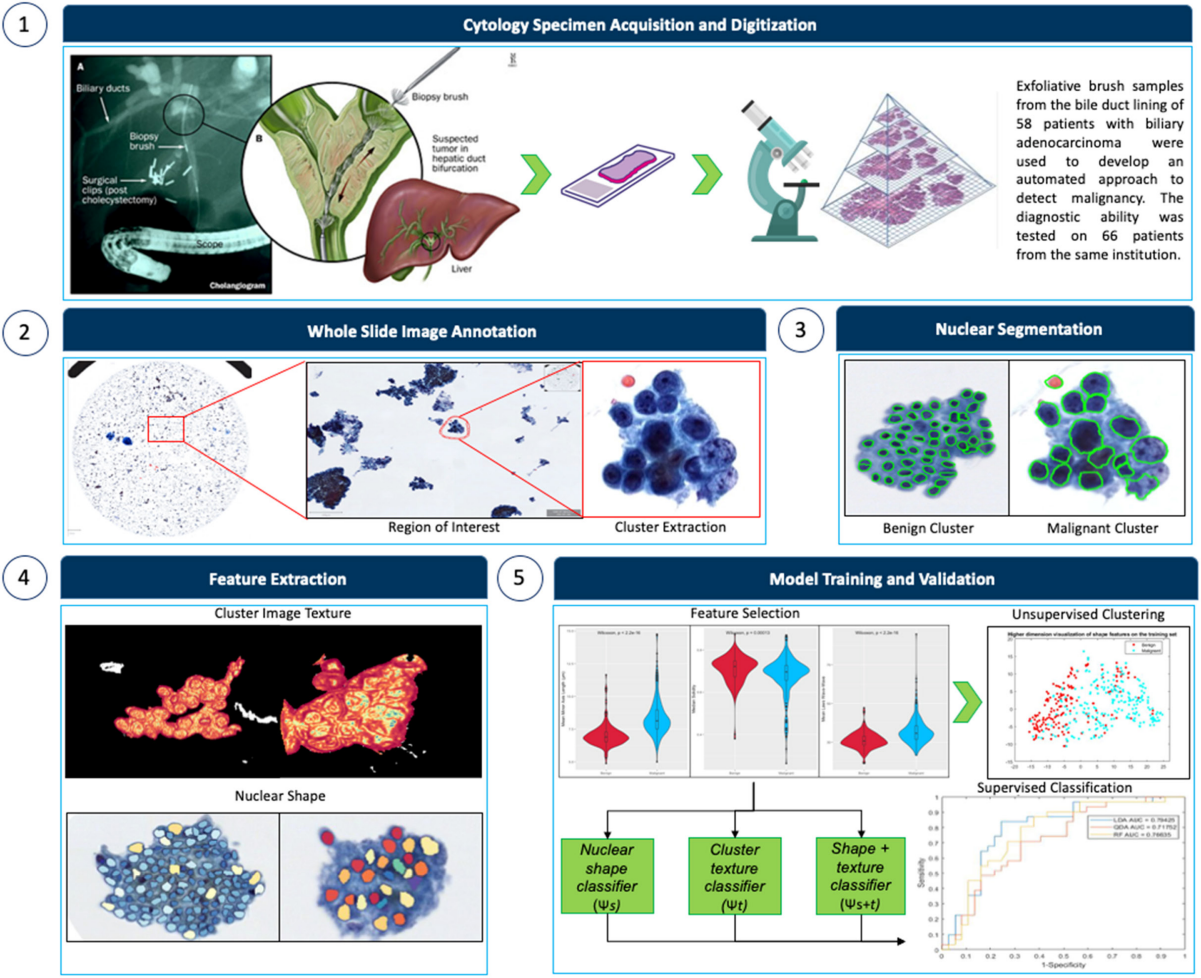
Overall methodological pipeline of this study. Benign and malignant clusters are annotated on whole slide bile duct brushings images. Regions of interest in the annotations are utilized to extract morphological features for pattern recognition and statistical analysis to differentiate benign and malignant morphologies by training machine‐learning classifiers on these annotated images

In this study, we investigated the role of computer‐identified imaging markers in improving the accuracy of pancreato‐ and biliary adenocarcinoma diagnosis from BDB cytology specimens through the use of morphological image analysis. We also evaluated the use of handcrafted machine‐learning classifiers, including linear discriminant analysis, quadratic discriminant analysis, and random forest, to predict malignancy on cytology specimens. The overall methodological pipeline of this study is illustrated in Figure 1.

## 2 MATERIALS AND METHODS

### 2.1 Data set description

**FIGURE 2:**
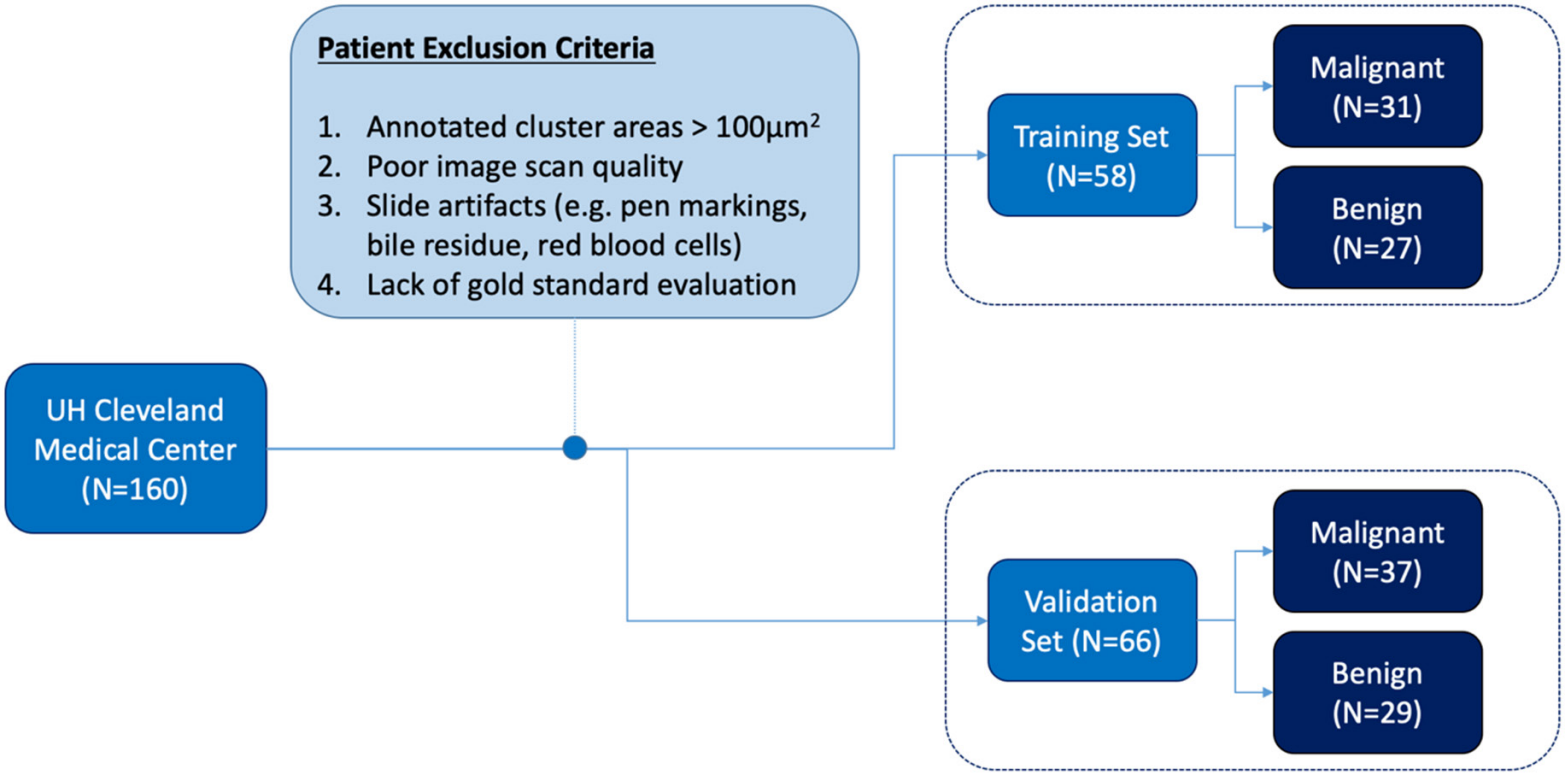
Criteria for including and excluding patients from the cohort collected from UH Cleveland Medical Center. The remaining 122 patients were allocated into training and testing sets with the objective of maintaining a balance of malignant and benign cluster images between all patients

Two sets of digitized BDB WSIs were used in this study, both from the University Hospitals Cleveland Medical Center (UHCMC). Slides from both cohorts were prepared using liquid‐based ThinPrep cytology and Papanicolaou staining. The first set of images was delegated as the training set (*S*_T_), which contained 66 WSIs corresponding to 66 patients with BDBs collected between 2011 and 2018. Each patient was diagnosed on cytology as either malignant or benign with the clinical–pathological follow‐up. The follow‐up was based on clinical progression, imaging, and histology wherever available. Cases that were diagnosed as benign by both cytology and machine were labeled benign based on the 2‐year clinical and radiological follow‐up. Cases that did not have any follow‐up were excluded from this study. WSIs from each cohort with poor scan quality and slide artifacts were removed from this study. The quantitative breakdown of patients for *S*_T_ before and after the exclusion process is shown in Figure 2. After excluding patients without confirming the clinical follow‐up, regardless of the availability of diagnosis on cytology, 58 patients remained in the training cohort, 31 were confirmed with the presence of adenocarcinoma, and the remaining 27 were classified as having benign lesions.

The second set of images corresponded to 94 patients (66 after exclusion) and was used as an independent validation set (*S*_V_) to test the robustness and accuracy of the trained machine‐learning classifiers. *S*_V_ contained benign and malignant as well as atypical cases based on the cytological diagnosis. Reactive cases were considered benign, whereas suspicious cases were folded into malignant ones for statistical purposes. All other cases which could not be classified as benign or malignant were included in the atypical category on the cytological diagnosis. All diagnoses of *S*_V_ on machine‐based predictions were either in the benign or malignant categories based on the clinical–pathological follow‐up, whereas cytological categories include benign, malignant, and atypical cases. Each diagnostic category for cytology, clinical follow‐up, and machine prediction is shown in Table 2. *S*_V_ comprised 37 malignant and 29 benign patients after the exclusion process.

### 2.2 Slide digitization, annotation, and preprocessing

**FIGURE 3:**
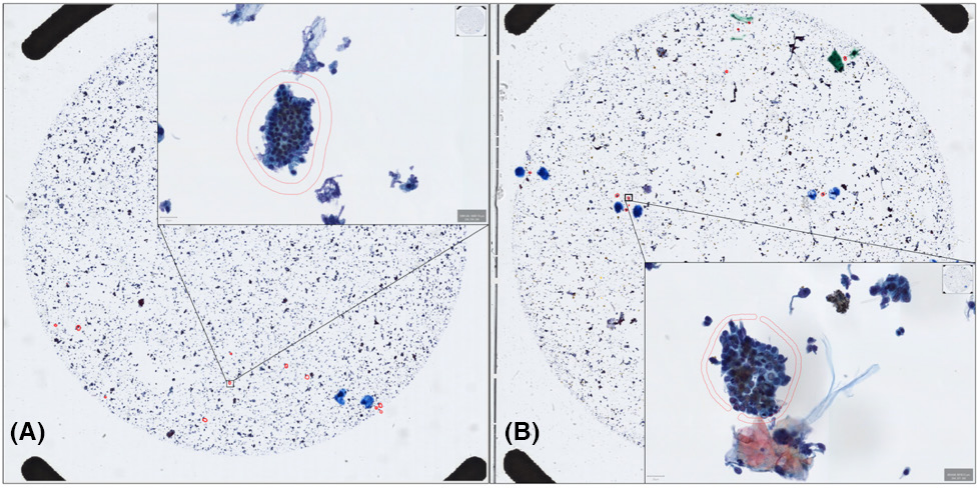
(A) Benign and (B) malignant whole slide image of biliary tract brush cytology specimen under centrifugation and magnified under 40×. Pathologist annotations of benign and malignant clusters are indicated in red markings

Each brush specimen slide was magnified at 40X using the Ventana iScan HT microscope and digitized to generate a WSI for each patient. Z‐plane settings were not incorporated during the digital scanning for each slide, thus each slide was viewed and scanned under the same focal length. Computerized diagnosis of each patient was done by identifying selected cell clusters on each WSI. For *S*_T_, an expert cytopathologist annotated candidate clusters for image‐based feature extraction based on the visual quality of clusters present on each WSI (see Figure S1). Based on their domain expertise and discretion, the pathologist selected clusters based on the inclusion of cellularity variety, prominence of nuclear boundaries, and prominence of nucleoli. Clusters on each benign patient WSI that were affected by scan quality, artifacts, or were <100 μm^2^ were excluded from the feature extraction process. Five clusters chosen from the area encircled by pen markings on benign WSIs on *S*_T_ were extracted for image analysis. Similarly, for patients with pancreaticobiliary, five malignantly identified clusters per WSI were annotated as the ground truth for our supervised approach to classification. Open‐source software for WSI processing, QuPath, was used to generate the annotations by encircling clusters of interest on the WSI. Cell cluster images were generated by obtaining the bounding box of each annotation, generated by the coordinates of the annotation. Figure 3 illustrates how the slide images were viewed and annotated.

*S*_T_ containing annotations of cell clusters extracted from 58 WSIs contained both the region of interest (the cluster itself) and the slide background. The background noise was removed by filtering pixels with a grayscale intensity greater than a preset threshold from each annotation, determined by the average background intensity of the annotation. Smaller objects, such as stain artifacts, pen markings, bile residue, and individual cells, with an area less than a predefined threshold, were digitally removed from the annotation as well. Contrast enhancement was performed on each cluster by a histogram equalization method to better visually separate the nuclei from the cytoplasm. Performing contrast enhancement also allows for the improvement of edge‐based segmentation methods.

### 2.3 Automatic detection of cell clusters on whole slide images

For novel images unannotated by the pathologist in *S*_V_, WSIs were deconstructed into 1000X1000 pixel area patches. Although it would be ideal to extract every cluster visible on a slide image, patches that contained over 40% of the background image area were digitally removed from the selection process. The clusters in the remaining patches were then identified by filtering background pixels with grayscale intensity greater than a predefined threshold. Clusters were filtered through the same process described for *S*_T_. Of the 66 patients in *S*_V_, 275 clusters were extracted from the 37 confirmed malignant patients and 170 clusters from the 29 confirmed benign. Fifty cluster images were randomly selected for quality assurance by the cytopathologist.

### 2.4 Nuclear segmentation on cell clusters

Nuclear segmentation was performed on all cell clusters in *S*_T_ and *S*_V_, to delineate the boundary pixels of nuclei using a watershed method.^18^ A binary mask was then generated for each cell cluster image by using the segmented nuclei boundaries outlining the shape of the nuclei in each cell cluster. The watershed algorithm split clustered objects by first identifying the regional minima in a negative distance map of a binary array and then determines boundaries by outwardly growing from regional minima until separating boundaries are reached.^18^

### 2.5 Extraction of morphological features

#### 2.5.1 Aggregate cluster texture features

Image texture features were extracted in MATLAB from the entire area of cell clusters to obtain a global quantitative assessment of potentially subvisual diagnostic signals, which includes the interaction between nuclei and cytoplasm. Before any texture‐based features were extracted, the local effects of illumination were removed by moving a 15X15 window on each cell cluster image iteratively for each pixel and subtracting the local average for each window from the original image. Four different families of texture features were extracted subsequent to the normalization of illumination. The specific parameters for each texture‐related feature are described in the Appendix S1. The specific classes of texture features extracted included Gabor, Law's, Haralick, and CoLlAGe (Co‐occurrence of Local Anisotropic Gradient Orientations) feature families.

The Gray‐Level Co‐occurrence Matrix (GLCM) measures the joint probability of occurrence of any given pair of intensity values in a local window for each pixel. Second‐order statistical measures were extracted from this matrix, called Haralick features, which describe metrics of pixel intensity contrast, entropy, energy, homogeneity, and correlation. The Haralick feature family contained 13 unique 5 × 5 kernels, quantized to 64‐level intensity.^19^ CoLlAGe features employ similar kernels as the Haralick feature family but are combined with the Histogram of Oriented Gradients (HOG) feature descriptors. Gradient magnitudes were computed for each pixel, and then local dominant orientations are computed by singular value decomposition. The most significant orientations were then determined by computing second‐order statistics similar to the Haralick features.^10^ As with the Haralick kernels, 13 unique 5 × 5 CoLlAGe kernels were convolved with each input image.

Gabor responses were computed from the convolution of a sinusoidal‐modulated Gaussian kernel function.^20^ These filters were used to extract dominant‐oriented textures within the cell cluster. A total of 48 Gabor features were extracted across different frequencies and orientations for each cell cluster image and are listed in the Appendix S1.

Law's texture features were computed by convolving a set of 1D line kernels with each cell cluster image. These features aim to identify the level (L), edge (E), spot (S), wave (W), and ripple (R) patterns. A set of 25 unique 5 × 5 kernel response maps were computed by taking the outer product for each possible pair of vectors.^21^ The parameters of each line kernel are described in the Appendix S1.

Each family of texture features was computed at a pixel level. To obtain global‐level feature values for each cluster, five statistical measures were computed for each texture descriptor for each cluster: mean, median, standard deviation, skewness, and kurtosis. Thus, a total of 495 texture features were extracted from 99 kernel responses across the entire cell cluster regions. The texture features were evaluated at an aggregate level on the entire cluster rather than on individual nuclei to investigate the potential interplay between nuclei and cytoplasm in malignancy.

#### 2.5.2 Nuclear shape features

Two‐dimensional shape properties of the nuclei were computed to serve as the basis for differentiating morphologies of benign and malignant clusters. Forty shape features were extracted for each nucleus and were transformed into cluster‐level metrics by taking the mean, median, standard deviation, skewness, and kurtosis of nucleus‐level features at a cluster level. The nuclei‐to‐cytoplasm (N:C) ratio was also computed per cluster by dividing the total nuclear area by the nonnuclear area. Thus, a total of 41 shape features were extracted per cluster and used in the subsequent classification experiments. A more exhaustive description of the features extracted is listed in Table S1. Shape features were computed based on measurements depicted in Figure S2.

### 2.6 Statistical analysis

All 41 shape features and 495 texture features extracted from each cluster image were ranked on class discriminability using the Wilcoxon rank‐sum (Mann–Whitney) test,^22^ the two‐sided t test, and the minimum redundancy maximum relevance (mRMR) optimization algorithm.^23^ The Wilcoxon test evaluates the difference in medians rather than means between two distributions. It does not assume normally distributed benign and malignant classes or any prior known distributions. The mRMR scheme was chosen to identify and prune statistically dependent textural features while selecting features that would provide optimized classification performance given these constraints. Top‐ranked features were deemed as statistically significant features (*p* = 0.05) for the Wilcoxon rank‐sum and t tests that were the most frequently selected on each fold during 100 iterations of cross‐validation on *S*_T_ and a Pearson correlation coefficient threshold of 0.7 for mRMR. Feature selection and classification steps were all performed in MATLAB.

The classifiers included a linear discriminant analysis (LDA), quadratic discriminant analysis (QDA), and a bagged random forest (BaggedC4.5). The choice of classifiers was based on the range of complexity from a simple, linear model (LDA) to a highly nonlinear, highly complex model (Bagged C4.5). The cross‐validation performance of each classier was measured in terms of area under the curve (AUC) of the receiver operating characteristic (ROC) curve, accuracy, sensitivity, and specificity. To avoid potential bias and class imbalance during training, a multi‐expert system was implemented in each fold, where parallel classifiers are trained on an equal number of both classes.^24^ The number of samples in each fold was calculated as a function of the state of the class imbalance. For any given benign class with size *b* and malignant class with size *m*, where *b < m*, the first classifier was trained with *b* benign examples and *b* randomly sampled malignant examples. The second classifier was then trained with (*m – b*) benign examples randomly sampled from *b* and (*m – b*) malignant examples, where (*m – b*) is the size of the complement set of malignant examples. The performance was averaged to compute overall balanced metrics.

The five most diagnostic aggregate cluster texture features selected from each of Wilcoxon, t test, and mRMR were trained on LDA, QDA, and random forest classifiers and evaluated on AUC, sensitivity, and specificity with 95% confidence. Each classifier was then used to validate the diagnostic ability of the features selected on *S*_T_ by computing a probability of malignancy for each cluster on *S*_V_. The AUC was computed by varying the threshold of probability of malignancy, which describes the classifier's general ability to diagnose patients correctly over a combination of sensitivity and specificity operating points.

## 3 RESULTS

### 3.1 Experiment 1: Evaluating the role of aggregate texture of cell clusters to distinguish malignant and benign phenotypes

**FIGURE 4:**
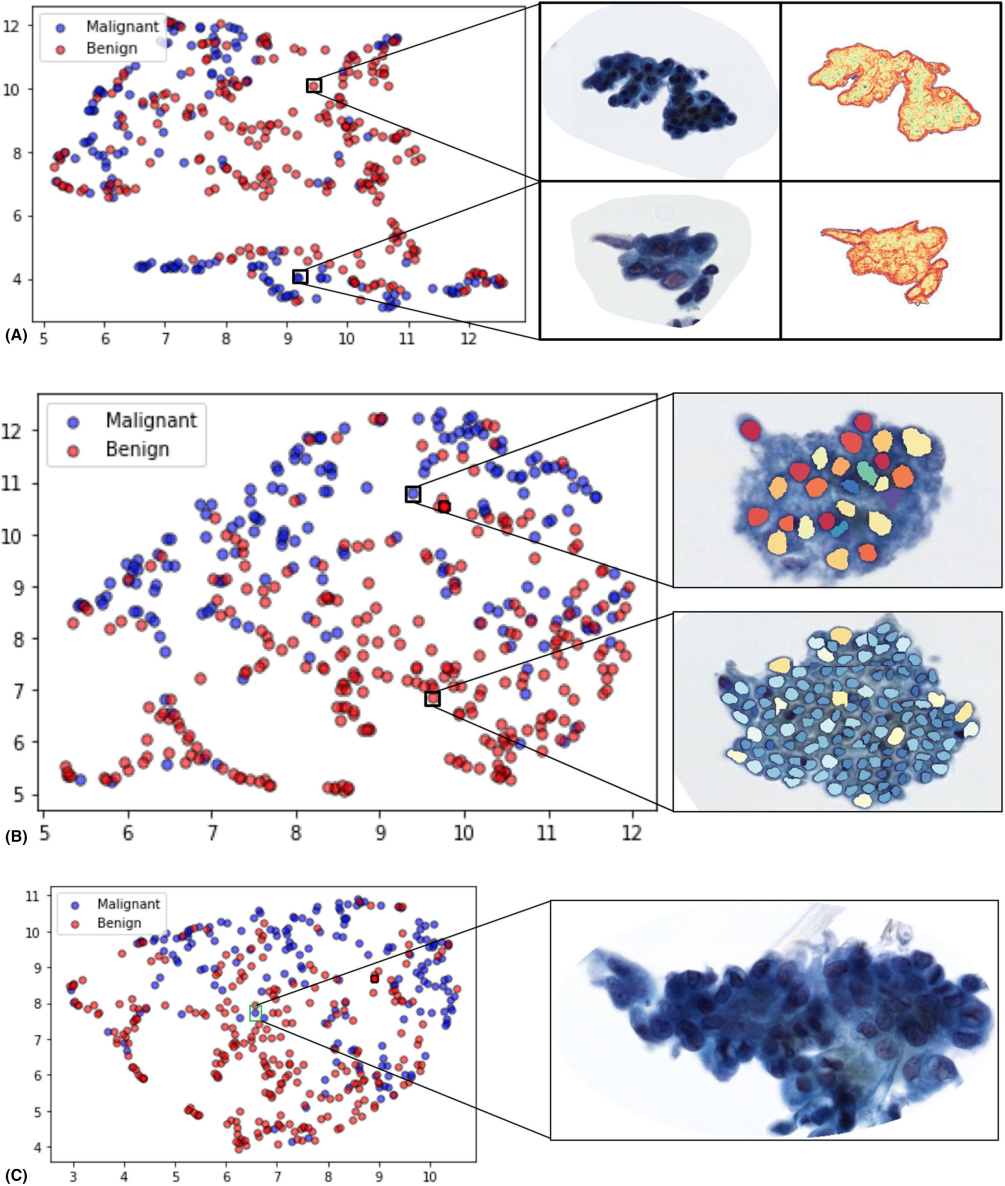
(A) Uniform manifold approximation projection (UMAP) visualization for Experiment 1 indicates discrepancies in aggregate texture between benign (top) and malignant (bottom) clusters using the top five texture features selected from the *t* test. The top feature (CoLlAGe information measure 1) is visually depicted as a heat map on the cluster. Red regions represent a high magnitude of contrast entropy around the nuclei, more so around the perimeter of malignant nuclei, whereas blue regions indicate regions of lower contrast entropy toward the center of benign nuclei. (B) UMAP visualization for experiment 2 with the top five nuclear shape features selected by the *t* test. The overlaid color polygons on the nuclei represent the magnitude of difference between benign and malignant clusters for the minor axis length feature. (C) UMAP visualization for Experiment 3 demonstrates the improvement in cluster separation when combining all feature families. An outlier case (cholangiocarcinoma) that was not diagnosed correctly and did not cluster with fellow benign cases is shown, likely due to its atypical nature which makes nuclear segmentation difficult

The most prognostic feature set that best separated malignant and benign clusters was the LDA classifier with t test selected features, as shown in Table 1a, with an AUC of 0.85 ± 0.01, a specificity of 0.75 ± 0.01, and a sensitivity of 0.84 ± 0.04 on 100 iterations of three‐fold cross‐validation, as shown in Table 1a. These features included the mean Haralick information measure 2, standard deviation of CoLlAGe correlation, mean CoLlAGe correlation, mean intensity entropy, and median of contrast entropy. The benign clusters exhibit on average a smaller local textural homogeneity than their malignant nuclear counterparts. The mean information measure 2 correlation is higher in benign clusters, however, in malignant clusters, we observe greater variance and mean in the CoLlAGe correlation. Malignant clusters also exhibit on average greater intensity entropy and lower contrast entropy. The ability of the texture features to separate the two classes is best represented by the two‐dimensional visualization uniform manifold approximation projection (UMAP) visualization in Figure 4A. The UMAP transformation is a stochastic, nonlinear map that visually depicts the diagnostic ability for all the top features.^25^ The UMAP plot in Figure 4A, constructed in Python, represents a cluster of benign samples that form the inner core of the manifold, while the malignant samples form the periphery of the benign cluster. The formation of distinct clusters suggests that the malignant and benign clusters are separable by selecting a few prominent features.

**TABLE 1:** Classification performance of different classifiers and feature selection tests trained on 54 patients for (a) Experiment 1, (b) Experiment 2, (c) Experiment 3

	AUC	Accuracy	Specificity	Sensitivity
(a) Experiment 1				
Texture classifier				
LDA + wilcoxon	0.82 *±* 0.02	0.77 *±* 0.02	0.74 *±* 0.05	0.80 *±* 0.05
LDA + ttest	0.85 *±* 0.01	0.80 *±* 0.01	0.75 *±* 0.04	0.84 *±* 0.04
LDA + mrmr	0.80 *±* 0.02	0.74 *±* 0.03	0.77 *±* 0.05	0.72 *±* 0.08
QDA + wilcoxon	0.80 *±* 0.03	0.76 *±* 0.04	0.71 *±* 0.06	0.79 *±* 0.08
QDA + ttest	0.80 *±* 0.02	0.78 *±* 0.02	0.66 *±* 0.07	0.87 *±* 0.04
QDA + mrmr	0.77 *±* 0.03	0.72 *±* 0.04	0.71 *±* 0.08	0.74 *±* 0.08
BaggedC45 + wilcoxon	0.85 *±* 0.02	0.79 *±* 0.03	0.75 *±* 0.05	0.83 *±* 0.06
BaggedC45 + ttest	0.84 *±* 0.02	0.81 *±* 0.01	0.69 *±* 0.05	0.89 *±* 0.04
BaggedC45 + mrmr	0.85 *±* 0.01	0.79 *±* 0.02	0.76 *±* 0.05	0.81 *±* 0.05
(b) Experiment 2				
Shape classifier				
LDA + wilcoxon	0.84 *±* 0.01	0.80 *±* 0.01	0.83 *±* 0.04	0.78 *±* 0.04
LDA + ttest	0.85 *±* 0.01	0.81 *±* 0.01	0.80 *±* 0.02	0.82 *±* 0.03
LDA + mrmr	0.84 *±* 0.01	0.80 *±* 0.01	0.78 *±* 0.05	0.81 *±* 0.05
QDA + wilcoxon	0.81 *±* 0.02	0.77 *±* 0.01	0.76 *±* 0.05	0.77 *±* 0.04
QDA + ttest	0.80 *±* 0.02	0.76 *±* 0.02	0.75 *±* 0.06	0.78 *±* 0.06
QDA + mrmr	0.81 *±* 0.02	0.77 *±* 0.03	0.77 *±* 0.04	0.77 *±* 0.06
BaggedC45 + wilcoxon	0.84 *±* 0.01	0.79 *±* 0.01	0.76 *±* 0.05	0.81 *±* 0.04
BaggedC45 + ttest	0.84 *±* 0.01	0.79 *±* 0.01	0.78 *±* 0.03	0.80 *±* 0.03
BaggedC45 + mrmr	0.82 *±* 0.01	0.78 *±* 0.00	0.76 *±* 0.05	0.80 *±* 0.04
(c) Experiment 3				
Combined classifier				
LDA + wilcoxon	0.85 *±* 0.01	0.81 *±* 0.01	0.76 *±* 0.05	0.84 *±* 0.04
LDA + ttest	0.86 *±* 0.01	0.81 *±* 0.01	0.77 *±* 0.03	0.83 *±* 0.03
LDA + mrmr	0.83 *±* 0.02	0.78 *±* 0.02	0.75 *±* 0.04	0.80 *±* 0.05
QDA + wilcoxon	0.82 *±* 0.01	0.78 *±* 0.01	0.73 *±* 0.06	0.82 *±* 0.05
QDA + ttest	0.83 *±* 0.02	0.79 *±* 0.02	0.74 *±* 0.04	0.82 *±* 0.05
QDA + mrmr	0.82 *±* 0.02	0.78 *±* 0.03	0.73 *±* 0.06	0.82 *±* 0.06
BaggedC45 + wilcoxon	0.86 *±* 0.01	0.81 *±* 0.01	0.75 *±* 0.08	0.85 *±* 0.06
BaggedC45 + ttest	0.86 *±* 0.02	0.81 *±* 0.01	0.76 *±* 0.04	0.86 *±* 0.03
BaggedC45 + mrmr	0.87 *±* 0.01	0.81 *±* 0.01	0.79 *±* 0.05	0.82 *±* 0.03

*Note*: Three‐fold cross‐validation with 100 bootstrap replications was implemented using the most diagnostic texture and shape features.

Abbreviations: AUC, the area under the curve; LDA, linear discriminant analysis; QDA, quadratic discriminant analysis.

### 3.2 Experiment 2: Evaluating the role of nuclear shape to distinguish malignant and benign phenotypes

Similar to Experiment 1, the five most diagnostic nuclear shape features were selected using the three different feature selection schemes, and each set was trained on each of the three machine‐learning classifiers. The best training performance was observed on the LDA classifier with features selected from the t test, as shown in Table 1b, with an AUC of 0.85 ± 0.01, a specificity of 0.80 ± 0.02, and a sensitivity of 0.82 ± 0.03 on 100 iterations of three‐fold cross‐validation. The nuclear shape classifier performed similarly in terms of AUC, however, presented with 5% greater specificity and 2% lower sensitivity. The five features selected by the t test were the mean nuclear perimeter, mean solidity, median minor axis length, median solidity, and standard deviation of the nuclear perimeter. The UMAP plot in Figure 4B shows the separation ability between malignant and benign clusters via the top five nuclear shape features. For reference, a visualization of the magnitude of the minor axis length for a benign cluster was compared against a malignant cluster. In general, the benign clusters tend to have on average a smaller minor axis length and a greater solidity (more firm and rigid shape) compared with the malignant clusters.

### 3.3 Experiment 3: Evaluating the combination of nuclear shape and cluster texture features to distinguish malignant and benign phenotypes

The best five nuclear shape and aggregate cluster texture features from each of the above experiments (10 in total) were identified using each feature selection algorithm and subsequently used to train the same set of classifiers. The most accurate classifier was the LDA classifier with features selected from mRMR, as shown in Table 1c, with an AUC of 0.87 ± 0.01, a sensitivity of 0.79 ± 0.005, and a specificity of 0.82 ± 0.03 on 100 iterations of three‐fold cross‐validation. Combining the top features from each feature family resulted in an improvement in sensitivity of 2% from 75% to 77% and training AUC by 1% from 85% to 86%. The top five features selected from both shape and texture families were independently validated on *S*_V_. These pertained to the mean contrast entropy, standard deviation of CoLlAGe correlation information measure 1, mean solidity, mean intensity entropy, and median minor axis length. Figure 6 demonstrates the validation AUC of each classifier with features selected from mRMR, with LDA having the highest AUC of 0.79 on *S*_v_. The weighted score that represents the effect size on the outcome of machine diagnosis of each selected feature is shown in Figure S3.

Figure 4A illustrates benign and malignant cluster presentations and their corresponding contrast entropy feature maps. This feature includes measures of entropy in the computed gray‐level probabilities. The maps reveal that the nuclear boundaries are present with stronger edges compared with the benign clusters.

From Table 2, when selecting an operating point on the ROC with a threshold on the probability of malignancy of 30%, two patients were classified as false positives. Twelve patients were classified as false negatives, two of which were cholangiocarcinoma and could be explained due to bland morphology since both patients tested negative on cytology. Out of the total 15 cases with the atypical diagnosis on cytology, 10 were correctly diagnosed as malignant and one was correctly diagnosed as benign by machine diagnosis. The remaining four cases were benign on machine diagnosis but malignant on clinicopathological follow‐up. The sensitivity of cytology alone (calculated without atypicals) was 74%, whereas it was 46% when calculated with atypicals. However, with machine diagnosis, the sensitivity improved to 68% when atypicals were included and treated as nonmalignant false negatives. Within the atypical category, the machine‐learning model had a specificity of 100% within the atypical category as no false positives were identified, Additionally, 14 out of 17 patients with a malignant diagnosis on cytology were correctly predicted.

**TABLE 2:** Diagnostic results on *S*_*v*_

Cytological diagnosis	Machine diagnosis (*P*_mal_ = 0.3)	Clinical follow‐up		Total
		Benign	Malignant	
Benign	Negative	26	5	31
	Positive	2	1	3
Atypical	Negative	1	4	5
	Positive	0	10	10
Malignant	Negative	0	3	3
	Positive	0	14	14
	Total	29	37	66

## 4 DISCUSSION

The criteria for diagnosis of bile duct lesions have been well studied and standardized to provide cytopathologists with clear guidelines. The Iowa criteria devised in 1995 by Cohen et al. proposed a set of primary malignancy indicators, which reaffirmed the presence of nuclear molding, chromatin clumping, and increased nuclear‐to‐cytoplasmic (N:C) ratio.^14^ If none of the above were present, secondary criteria would be used, including anisonucleosis, nuclear irregularity, and relative nuclear enlargement.^14^ The Boston Criteria devised in 1998 by Renshaw et al. suggested that certain presentations in cell clusters were associated with malignancy, including nuclear molding, disorderly spatial arrangement of cells (low cellular polarity), and chromatin clumping.^16^ A selected few have performed analysis on WSIs of atypical and suspicious BDBs. Collins et al. manually selected 10 well‐visualized clusters and evaluated computational features such as individual nuclear area, nuclear count per cluster, N:C ratio, and nuclear size differential.^17^

In this study, we presented a computerized cytomorphological and textural analysis of cell clusters extracted from WSIs of digitized BDBs to quantify the morphological differences between benign and malignant presentations. We were able to identify five nuclear shape and five aggregate texture morphological features that best distinguish benign clusters from malignant clusters in 58 patients and successfully validate those features in 66 patients. The intent was to show that the sensitivity of diagnosis for BDBs can be improved by using computerized textural analysis of a combination of nuclear shape and texture features.

**FIGURE 5:**
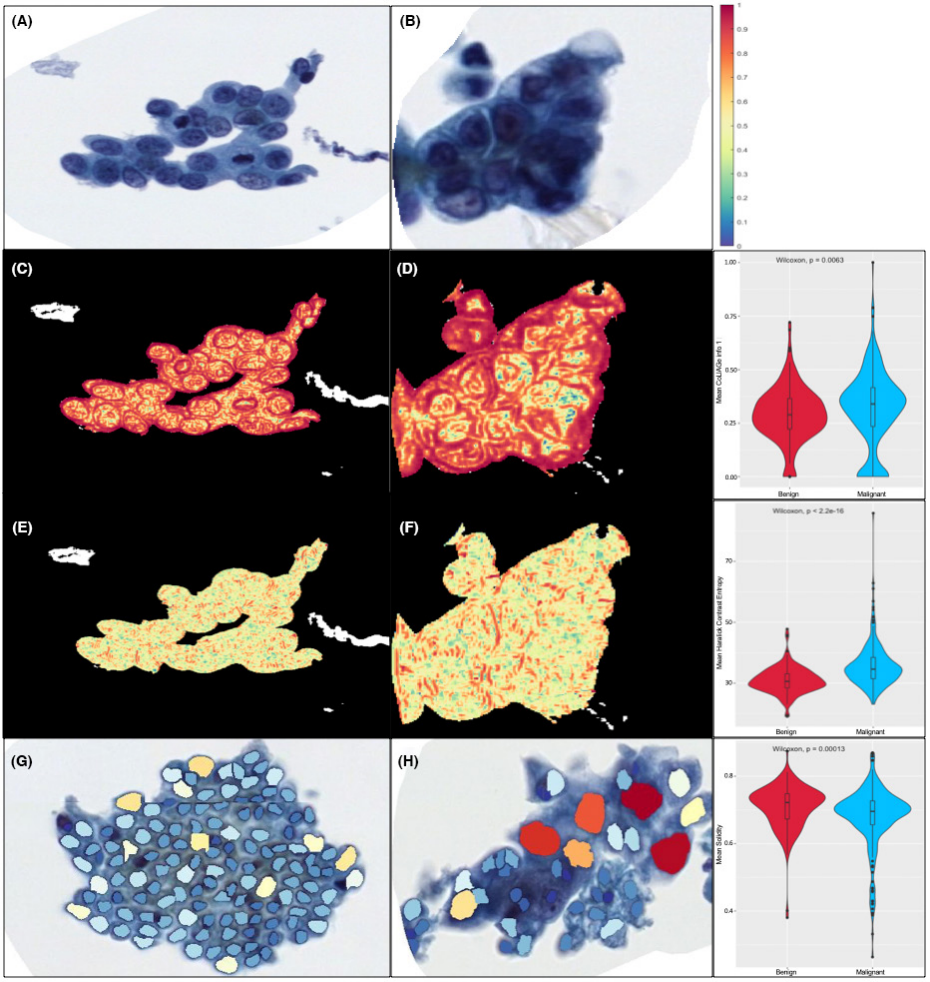
(A) Benign and (B) malignant cell cluster image magnified at 40×. Feature heat map and distribution visualization for the top texture feature from Experiment 3 indicating discrepancies in aggregate texture between (c) benign and (D) malignant clusters. Mean Haralick contrast entropy depicted on (E) benign and (F) malignant clusters. Feature overlay and distribution for the top shape feature mean solidity for (G) benign and (H) malignant clusters

The most frequently selected feature was the “Mean CoLlAGe information measure 1” feature, visually depicted with magnitude heat maps in Figure 5C,D, suggesting that larger areas of highly correlated pixels reflect regions of textural homogeneity. These regions of larger energy sums on the heat maps represent textural homogeneity, likely reflecting regions of dense chromatin distribution and showing evidence of nuclear overcrowding. The malignant cluster in Figure 5D shows more regions of stronger correlation than the corresponding benign clusters in Figure 5C, in turn, reflecting more homogeneous nuclear regions. This appears to suggest evidence of hyper‐ and hypochromasia, one of the primary criteria of malignancy.^2^ The presence of nucleoli is shown to be more prominent here, highlighted by the yellow regions inside the nuclei encircled by the darker red regions. Thus, the distribution of these dark spots contributes to poorer local pixel intensity correlation and a higher deviation in correlation for malignant clusters. Regions of higher correlation in the malignant cytoplasm may potentially be reflecting abnormal secretion of proteins. Particularly around the edges of malignant nuclei, as shown in Figure 4A, the presence of more dominant red edges packed around nuclear boundaries due to greater textural correlation in malignant clusters might reflect the presence of marginal chromatin in malignant phenotypes and the presence of three‐dimensional nuclear overcrowding of malignant nuclei found on the BDB specimens as a result of erratic growth of nuclei on top of one another. In contrast to malignant nuclei, benign nuclei are arranged in sheets, in turn reflecting a more orderly arrangement of nuclei. The blue regions (area of a lower likelihood of similar gray level pixel probabilities) within the malignant nuclei are likely a consequence of the denser distribution of chromatin within the nuclei. The yellow regions in the benign cluster represent the cytoplasmic region, in turn reflecting a sparse distribution of cytoplasmic contents through the cytoplasm. Conversely, the cytoplasm in malignant clusters presents with a higher degree of correlation, which in turn suggests a more heterogeneous and disorderly distribution of cytoplasmic contents.

**FIGURE 6:**
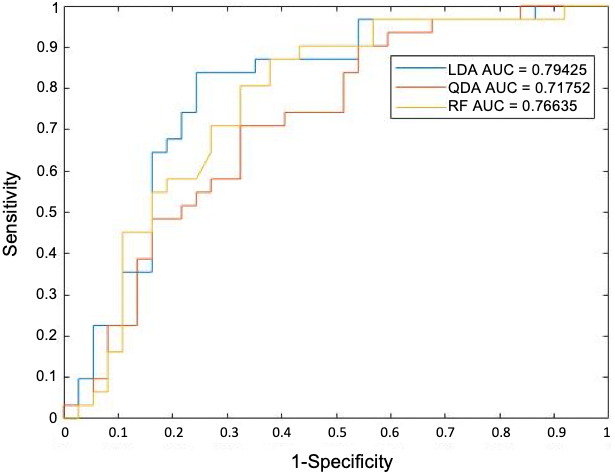
Receiver operating characteristic curves for each classifier trained on a mix of five shape and texture features selected by mRMR. Each classifier was validated on 66 patients

The next most frequently selected diagnostic feature in Experiment 3 was the standard deviation of the CoLlAGe information measure 1, possibly reflecting the fact that malignant clusters have a larger variance in the magnitude of local textural correlations. The third most frequently selected feature in Experiment 3, the mean solidity of nuclei depicted in Figure 5G,H, is a nondimensional metric that measures the irregularity of polygons, of benign nuclei is much higher than that of malignant nuclei. This could indicate that the shape of benign nuclei is in general more circular/ovular than their malignant counterparts.

In addition, the minor axis length of each nucleus of a benign cluster was in general lower compared with a malignant cluster. Not only is the mean value of the minor axis lengths lower than the corresponding benign clusters, but the variability in these quantitative metrics is much smaller compared with the malignant cluster. The larger minor axis length could reflect the fact that malignant nuclei are generally larger in volume and have a larger aspect ratio than their benign counterparts.

Our combined machine‐learning classifier achieved an AUC of 0.79 while selecting an optimum operating point on the ROC with a sensitivity of around 68% and specificity of 93%. Traditionally, the specificity of diagnosis of BDBs is high (up to 99%), however, the sensitivity is very poor (can reach as low as 44%),^26^ meaning that the rate of false negatives is problematically high. The diagnosis of BDBs remains a challenge for the pathologist due to the local effects of strictures and low cellularity in clusters, which tend to be insufficient in making a diagnosis. A recent study by Kuzu et al. found that the sensitivity can reach up to 62.4% on brush cytology.^27^ The reason for poor sensitivity is that atypical clusters can neither be definitively classed as adenocarcinoma nor benign and leads to uncertainty in management.

The sensitivity that we report is considerably higher than the current rate provided by standalone brush cytology. For the 12 patients who were classified as false negatives, one such case is illustrated in Figure 4C, which belongs to a cluster of cholangiocarcinoma cases that did not cocluster accordingly with other malignant cases on the UMAP. The machine‐learning model had a specificity of 100% within the atypical category as no false positives were identified, which is a particularly encouraging result for these difficult‐to‐interpret cases since unnecessary treatment would not be required for an otherwise malignant diagnosis. Additionally, 14 out of 17 patients with a malignant diagnosis on cytology were correctly predicted. Harbhajanka et al. supplemented cytomorphologic analysis with molecular profiling of postcentrifuged samples by targeted NGS analysis with a sensitivity of 93% and a specificity of 100%.^26^ The specificity of our classifier performed near this standard at 93% and did not require complex laboratory testing, destruction of tissue, or an expensive setup. The higher sensitivity of our approach compared with pathologists suggests that it could serve as an initial decision‐support tool in low‐resource settings. These findings appear to provide a biological rationale for the “hot” regions identified on the classifier‐generated heat maps. These findings were also corroborated by a team of pathologists and are briefly summarized in Table S2.

A possible explanation for the increase in false‐negative errors with our computerized image classifier approach is the discoloration in stains due to variability in chromatin distribution and staining errors. This is particularly prevalent in the benign cytology category, where 5 out of 31 patients were incorrectly classified. These various preanalytic sources of variation could potentially have affected the spatial arrangement of colors detected by the texture features. Nuclear overlapping, identified as one of the malignancy criteria, is also present in benign clusters and also contributed to some of the false‐positive errors due to the folding of cells on top of each other.

A considerable fraction of medical literature using machine‐learning‐based image analysis has relied on DL approaches to examine images and extract quantitative morphological features.^28^ DL methods have become extremely popular in computational pathology since they are unsupervised feature‐generation approaches using different neural network architectures. However, the opacity of the generated features has resulted in these methods being labeled as “black‐box” approaches, a potential bottleneck to the clinical application.^29^ In contrast, our hand‐crafted approach relies on a foundation of biologically motivated and well‐defined cytological criteria. As suggested by recent review articles,^28^, ^30^ these hand‐crafted feature‐based approaches might be more amenable to clinical translation and adoption compared with black‐box‐based approaches.

The main limitations of our study were the small sample size of 124 patients (across both data sets after quality control), lack of independent validation across multiple institutions, lack of dedicated human‐machine comparison, lack of class balance in the atypical category of cytology, and the lack of head human‐machine integration in a clinical setting. The ground truth annotations that were manually defined as a time‐consuming process for the cytopathologists. However, the approach presented in this work could potentially provide a method for potentially helping to triage obviously benign cases, and more importantly, to assess atypical patients with high specificity, hence helping to improve the efficiency of the diagnosis of BDB specimens, a process that could often involve hours of examination and consultation for a pathologist for a single patient.

## 5 CONCLUSION

In this study, we demonstrated that nuclear shape and cell cluster texture features can automatically distinguish benign clusters and adenocarcinoma on BDB cytology images with 68% sensitivity on benign, atypical, and malignant cases and 100% specificity on atypical cases. We identified a set of diagnostic features that could discriminate the two potential diagnoses. With additional independent validation, the approach could serve as decision support or triage tool for cytopathologists specializing in BDB cytology.

## AUTHOR CONTRIBUTIONS

Shayan Monabbati: Conceptualization, formal analysis, software and writing – original draft. Patrick Leo, Kaustav Bera: Methodology, writing – review and editing. Anant Madabhushi: Methodology, investigation, project administration, resources, funding acquisition, writing – review and editing. Claire Michael, Behtash Nezami, Aparna Harbhajanka: Data curation, methodology, writing – review and editing.

## ETHICAL APPROVAL STATEMENT

This article does not contain any studies involving human participants performed by any of the authors. This study was approved by the Institutional Review Board SpartaIRB under the ID: STUDY20200232: Deep learning to stratify borderline bile duct specimens. The collection of de‐identified digitized whole slide images and the analysis was approved under UH IRB 02–13‐42C. The requirement for informed consent was waived by the IRB since this was a retrospective de identified study.

## Supporting information


Appendix S1

## Data Availability

The data that support the findings of this study are openly available in GitHub at https://github.com/ccipd/.
