# Supplementary material for: Automated analysis of computerized morphological features of cell clusters associated with malignancy on bile duct brushing whole slide images
Source: Cancer Med. 2022 Oct 24;12(5):6365–78. doi: 10.1002/cam4.5365 (PMC10028025; doi:10.1002/cam4.5365)
Supplement: Supplementary file 1 — Appendix S1 [file CAM4-12-6365-s001.docx]

## Supplemental Materials


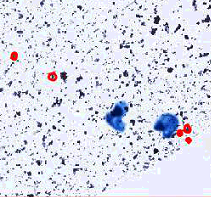


*Supplemental Figure 1 - A sample patch of a WSI for a negative patient. Annotations from the cytopathologist are indicated in red*

Laws’ Texture Feature Parameters:

Level (L5): [1 4 6 4 1]

Edge (E5): [-1 -2 0 2 1]

Spot (S5): [-1 2 0 -2 1]

Wave (W5): [-1 2 0 -2 1]

Ripple (R5): [1 -4 6 -4 1]

Gabor’s Texture Feature Parameters:

Spatial frequencies: {0, 2, 4, 8, 16, or 32}

Orientation: θ = 0, π/8, π/4, 3π/8, π/2, 5π/8, 3π/4, 7π/8

Supplemental Table 1: Nuclear Shape Properties:

| **Select 2D Shape Features** | **Measurement** |
| --- | --- |
| Area | μm^2^ |
| MajorAxisLength of the elliptical bounding box of annotation | μm |
| MinorAxisLength of the elliptical bounding box of annotation | μm |
| Eccentricity of the elliptical bounding box of annotation | $\sqrt{1-\frac{minoraxis}{majoraxis}}$ |
| Orientation of major axis length w.r.t horizontal | radians |
| Equivalent Diameter | $\sqrt{majoraxislength*minoraxislength}$ |
| Solidity | $\frac{area}{area of surrounding convex hull}$ |
| Perimeter | μm |


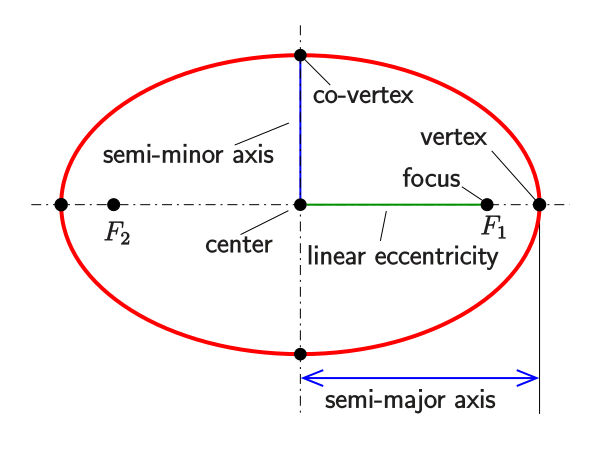


*Supplemental Figure 2 – Geometrical properties of an ellipse. A bounding ellipse is created around each nucleus to compute 2D shape features.*

Supplemental Table 2: Biological interpretation of top cytological imaging features

| Machine-selected Feature | Description on Cytology |
| --- | --- |
| Mean CoLlAGe info measure 1 | Textural homogeneity suggests evidence of hyper- and hypochromasia and nuclear overcrowding |
| Mean Haralick Contrast Entropy | Evidence of marginal chromatin distribution around nuclear boundaries |
| Std of CoLlAGe info measure 1 | Larger variance of cytoplasmic content and chromatin distribution in malignant nuclei |
| Mean Solidity | Benign nuclei are more circular and rigid in nuclear geometry |
| Mean Minor Axis Length | Malignant nuclei tend to have smaller minor axis lengths, describing less circular nuclear geometry |


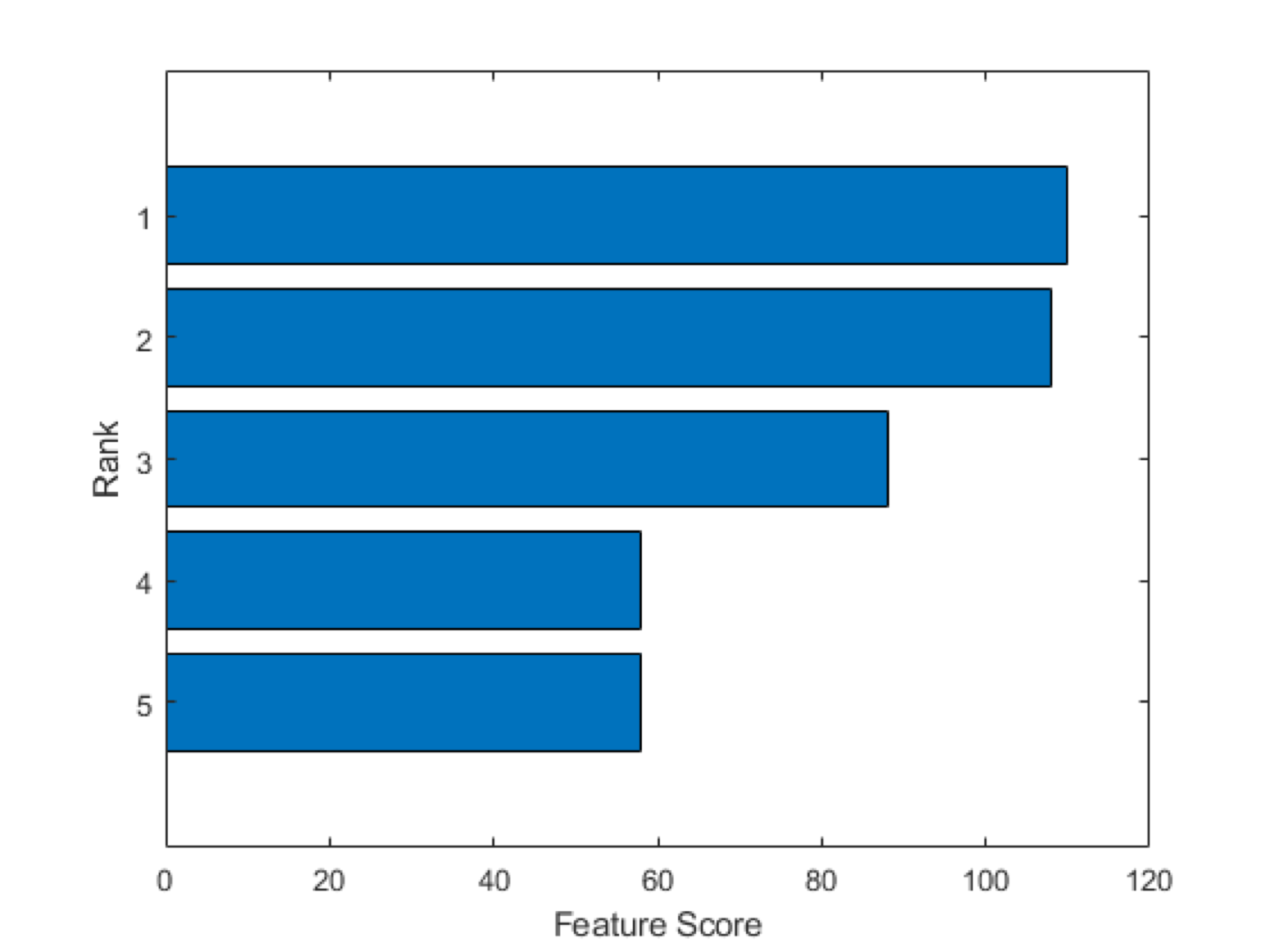


*Supplemental Figure 3 - Feature ranking for top 5 cytological imaging markers selected by mRMR test across all features. Feature score represents the effect size each feature has on the classification process. The names of each feature are according to the order in which they appear in Supplemental Table 2.*
